# Morphometric analysis of the incisive canal and foramen using CBCT: a cross-sectional study of the Saudi subpopulation

**DOI:** 10.7717/peerj.21701

**Published:** 2026-09-28

**Authors:** Hassan Ahmed Assiri, Abdullah Alqarni, Ali Mosfer A. Alqahtani, Ghaliah Alfwaih, Khulud Ali Alshehri, Budur Khalid Alsaanah, Nada Alshyarba, Taif AlQarni, Bushra Alshahrani, Maha Almansour, Rala Ebrahim Alalmai, Mohammad Shahul Hameed

**Affiliations:** 1 Department of Diagnostic Sciences and Oral Biology and Periodontology, King Khalid University, Abha, Asir, Saudi Arabia; 2 King Khalid University, Abha, Asir, Saudi Arabia

**Keywords:** Incisive canal, Nasopalatine canal, Incisive foramen, Cone beam computedtomography, Maxilla, Anatomic structures

## Abstract

**Background:**

The incisive canal (IC) and foramen are key anatomical structures in the anterior maxilla that provide passage for the nasopalatine nerve and vascular branches between the nasal and oral cavities. This study aimed to identify the morphometric characteristics of these two structures in a group of individuals from Abha City, Saudi Arabia, using cone-beam computed tomography (CBCT).

**Materials and Methods:**

Among the CBCT archived images acquired between May and August 2025 (*N* = 282), 200 scans were selected using computer-generated simple random sampling for eligibility screening; 100 eligible scans were included for morphometric analysis. The shape, length, position, and angulation of the canal (relative to the nasal floor), as well as the shape and diameters of the foramen, were evaluated, and their associations with age and sex were assessed.

**Results:**

The funnel shape (32%) was the most frequently observed form of the IC, followed by the hourglass (21%) and cylindrical (19%) forms. The incisive foramen was predominantly oval-shaped (55%), with heart-shaped and triangular forms occurring less frequently. The average length of the canal was significantly (*p* < 0.001) higher in males (11.06 mm) than in females (9.28 mm). The mean buccopalatal measurement of the foramen was significantly (*p* = 0.001) higher in males (3.77 mm) than in females (3.06 mm). Most of the patients had a single canal with either one (58%) or two (23%) foramens. Most patients demonstrated mild to moderate angulation of the IC, regardless of age group or sex. Canal extension beyond the nasal floor was categorized as <7 mm, 7–9 mm, 10–12 mm, and >12 mm.

**Conclusions:**

Within the limits of this study, we offer a comprehensive analysis of the morphometric characteristics of the IC and foramen across different age groups and sexes in a Saudi Arabian cohort using CBCT. Identification of variations in position and shape will help in clinical practice, particularly when administering local anesthetic block injections during dental treatment.

## Introduction

The incisive canal (IC), also called the nasopalatine canal, is a vital anatomical structure in the anterior maxilla that carries branches of the sphenopalatine artery and the nasopalatine nerve (Bornstein et al., 2011). It passes from the nasal cavity into the oral cavity and opens at the incisive foramen located near the maxillary central incisors on the palatal side (Mraiwa et al., 2004). The IC and foramen develop during early facial embryogenesis as part of the formation of the primary palate. They arise at the fusion site of the paired maxillary processes and the frontonasal prominence, forming a passage between the nasal and oral cavities. As the palatal shelves grow and merge, bone forms around the area, creating the bony canal and foramen through which the nasopalatine nerve and vessels pass.

The diameter, length, and shape of the IC vary, carrying significant implications for the clinical anatomist. In implantology, accurate knowledge of the dimensions and position is essential to avoid neurovascular injury, bleeding, or implant failure. Dental implant placement in the anterior maxilla can damage the neurovascular structures located within the canal (Thakur et al., 2013). In orthodontics, variations in IC guide safe insertion sites, especially during mini-implant placement. Periodontal procedures, such as palatal flaps or grafting, and surgical endodontics also require careful consideration of the canal to prevent complications, such as bleeding, paresthesia, and compromised surgical outcomes.

Cone-beam computed tomography (CBCT) has been used in dentistry for over 20 years. This sophisticated modality can illustrate anatomical structures in three dimensions (3D) (Hassan et al., 2009) and address magnification and two-dimensional (2D) projection issues found in standard tomography, such as panoramic and periapical radiographs (Naitoh et al., 2009). Advanced imaging techniques, including CBCT, are used to obtain detailed morphometric measurements of the maxillofacial region (Bermúdez-Pérez et al., 2023). High-resolution oral and maxillofacial anatomical structures can be consistently accessed with CBCT, which enables visualization of 3D anatomical landmarks with a lower radiation dose than conventional computed tomography (CT) (Kajan et al., 2015). However, the use of CBCT should follow guidelines and be limited to indicated cases to prevent unnecessary patient exposure and investigation-related costs. CBCT investigations highlight the influence of age, sex, and race on IC morphometry, underscoring the importance of regional data in clinical decision-making (Couso-Queiruga et al., 2026). Using localized morphometric standards can help identify the optimal implant location, thereby minimizing surgery-related risks and improving outcomes in the anterior maxilla (Chatzipetros et al., 2023).

Recent evidence indicates the need to evaluate the IC across different groups, as variations in its anatomy may have clinical significance (Alshamrani, Tokhtah & Al-Omar, 2021). Population-specific CBCT data can provide valuable reference values to support safe and effective clinical planning. Numerous studies have demonstrated the use of CBCT for this purpose across different races and populations, including various regions of Saudi Arabia (Linjawi et al., 2021; Alasmari, 2023). To the best of our knowledge, no study has been conducted on the population of Abha City in the Asir region of Saudi Arabia. Therefore, in this study, we aimed to conduct a morphometric analysis of the IC and foramen using CBCT scans from residents of Abha City. The goal was to provide clinicians with evidence-based information, enhance the accuracy of diagnosis and treatment planning, and reduce surgical risks in the anterior maxilla by detailing every local anatomic variant.

## Materials and Methods

### Study design and settings

This retrospective cross-sectional study used anonymized, previously acquired CBCT records obtained from the institutional database of King Khalid University, College of Dentistry (KKUCOD). The study was conducted after receiving approval from the Research Ethics Committee at King Khalid University (HAPO-06-B-001) (approval number: KKU-28-2025-14). Since the research involved no direct patient interaction and used no identifiable patient information, informed consent was not required. Archived CBCTs were analyzed from May 2025 to August 2025. All CBCT examinations obtained at King Khalid University Dental College Hospital for the period May–August 2025 formed the sampling frame (*N* = 282). A list of eligible examinations was generated, and 200 examinations were selected using computer-generated simple random sampling for screening to ensure feasibility of manual morphometric measurement. After applying predefined eligibility criteria, 100 scans were included for morphometric analysis. Reasons for exclusion were recorded a priori including limited field-of-view truncating the canal/foramen, motion/metal artifacts obscuring the incisive canal, prior surgery/trauma or pathology involving the anterior maxilla. The eligibility criteria were as follows: CBCTs free from artifacts and from patients aged >18 years old who did not have pathological conditions affecting the anterior maxilla that could interfere with the visualization and morphometric analysis of the IC. Images of inadequate quality and those with a limited view that did not clearly reveal the canal and foramen were excluded.

### Sample size and power analysis

This retrospective cross-sectional analysis included all scans meeting the eligibility criteria within the randomly screened sample, yielding a total of 100 scans (53 men and 47 women). As no further eligible scans were available from the screened sample, an a priori sample size calculation was not performed. Instead, a precision-based post hoc assessment was conducted using the observed variability of the primary outcome measures.

For the length of the IC, the observed standard deviation (SD; 2.57 mm) in a sample size of 100 corresponds to a 95% confidence interval (CI) half-width of approximately ±0.51 mm overall (±0.68 mm for males and ±0.79 mm for females), indicating adequate precision of estimation. For two-sample comparisons (α = 0.05, 80% power), the minimum detectable differences were approximately 1.45 mm for canal length and 0.60 mm for the buccopalatal diameter of the foramen. Given the estimation-focused objective and the 0.20-mm voxel size of the CBCT scans, the available sample size was deemed adequate to address the study’s aims.

### Radiographic measurements

All CBCT scans were obtained using the Kavo OP 3D Pro (Kavo Dental, Biberach, Germany), and analysis was performed with the OnDemand3D software^®^ version 1.0 (Build 1.0.10.7462) (×64 Edition). The acquisition parameters were 90 kVp, 6.3 mA, and 4.5 s. The field-of-view and voxel size of the retrieved images were 8 × 15 cm and 0.20 mm, respectively.

### The shape and dimensions of the incisive foramen

The shape and dimensions of the foramen were analyzed in the axial plane. The shape was characterized as pike, clover, heart, or oval based on a previous study (Tlili, Abdallah & Amor, 2017) (Fig. 1). Other variations in shape have also been described when observed. The diameters of the foramen were measured in both the buccopalatal and mesiodistal directions, and the greatest dimension was recorded (Fig. 2). In addition, the number of foramens observed on the axial plane on the CBCTs was recorded.

**Figure 1:**
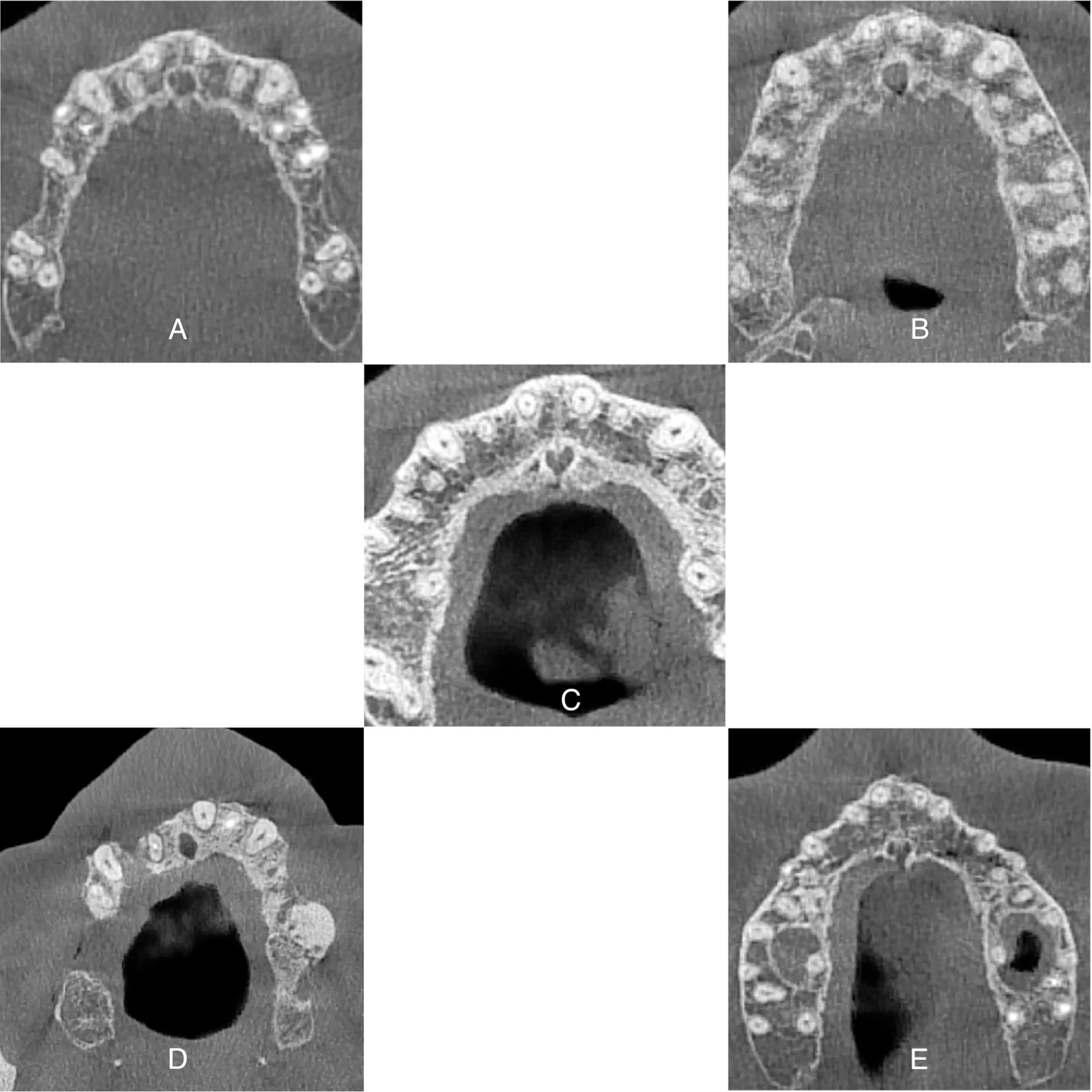
Incisive foramen shapes. (A) Oval shape. (B) Triangle shape. (C) Heart shape. (D) Pike shape. (E) Clover shape.

**Figure 2:**
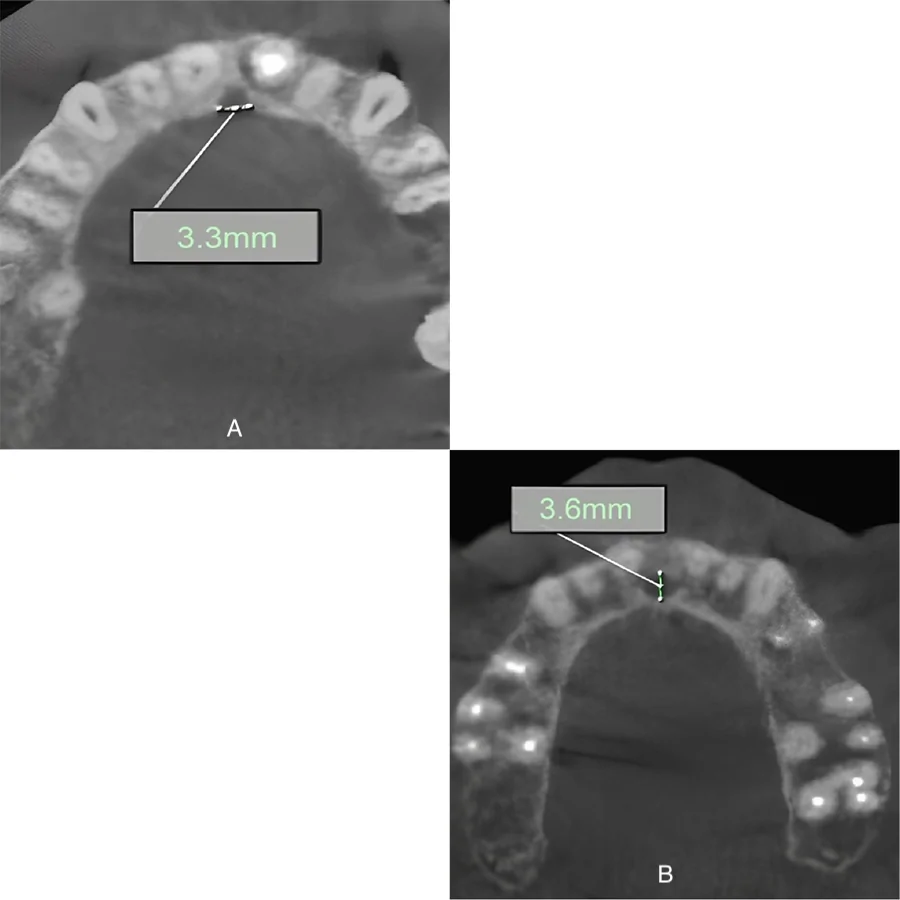
Diameters of the incisive foramen. (A) Mesio-distal diameter of the incisive foramen measured in the axial plane of CBCT. (B) Buccopalatal diameter.

### The shape and length of the IC

The shape and dimensions of the IC were analyzed in the sagittal plane using CBCT images. The shape was categorized as hourglass, inverted funnel, spindle, funnel, Y-shape, and cylindrical, as described previously (Tlili, Abdallah & Amor, 2017). (Fig. 3). The length was measured from the nasal opening down to the palatal opening. Regarding the position of the canal, we measured at its extension beyond the nasal floor to the palatal opening on the CBCT coronal plane, using the horizontal plane of the nasal cavity as a reference, and categorized it as follows: Type 1, where the canal extends below the nasal floor to less than seven mm; Type 2, where the canal extends 7–9 mm below the nasal floor; Type 3, where the canal extends 10–12 mm below the nasal floor; and Type 4, where the canal extends >12 mm below the nasal floor.

**Figure 3:**
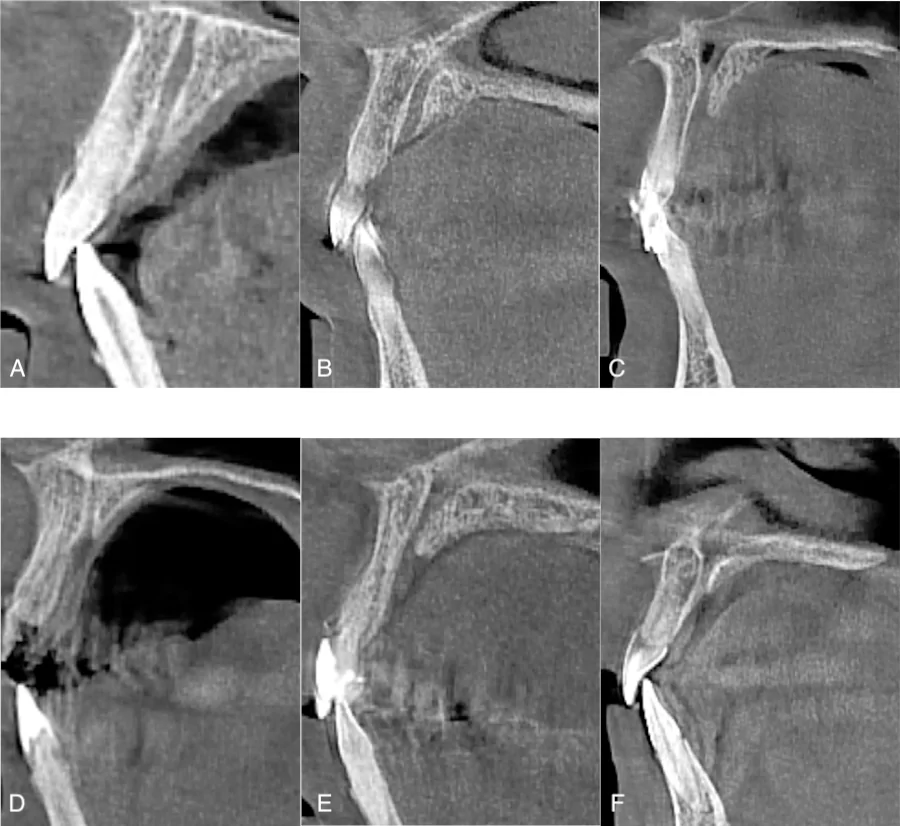
Incisive canal shapes revealed on the sagittal section of CBCT. (A) Spindle shape. (B) Cylindrical shape. (C) Hourglass shape. (D) Funnel shape. (E) Inverted funnel canal. (F) Y-shaped.

### Curvature of the IC

The curvature of the canal was measured based on the buccopalatal angulation in the sagittal plane of the CBCT at the point of greatest angulation (Fig. 4). The descriptive classification based on the degree of angulation was as follows: almost straight (>160°); mild (130–160°); moderate (110–130°); almost severe (<110°).

**Figure 4:**
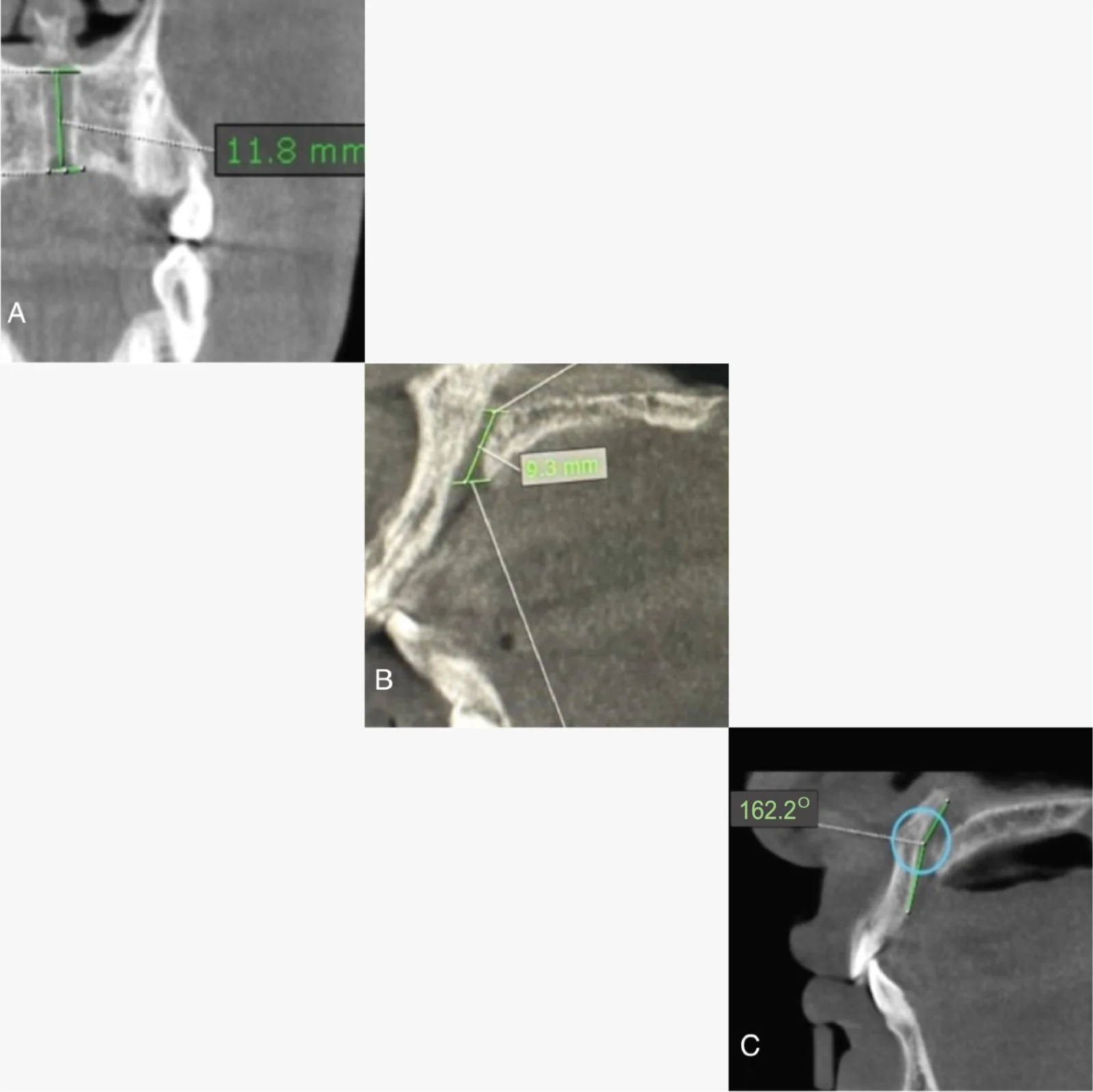
Measurement of the incisive canal length, position, and angulation on CBCT. (A) the incisive canal length is measured on the sagittal plane of the CBCT. (B) The position of the canal measured from the nasal floor to the palatal opening on the CBCT coronal plane, using the horizontal plane of the nasal cavity as a reference. (C) The canal angulation measured and revealed on the sagittal plane of CBCT.

### Observer’s reliability and agreement

After a collaborative training session with 10 pilot scans, two calibrated dentists performed independent measurements. Approximately 10% of the scans were re-evaluated, and both intra- and inter-examiner agreement were reported. Inter-observer reliability was assessed using a two-way random intraclass correlation coefficient (ICC; absolute agreement) for continuous variables and a weighted *κ* for categorical variables.

### Data collection and statistical analysis

A complete-case analysis was performed for variables with missing data. Regarding the categorization of the variables, the variables (age, canal extension, and canal angulation) were categorized to enhance clinical interpretability and facilitate comparison with previously published studies that have used similar groupings. In particular, age grouping allows assessment of clinically meaningful life-stage differences, while categorization of canal extension and angulation reflects anatomical classifications commonly used in CBCT-based morphometric studies. The clinical parameters, including age and sex, along with the radiographic parameters, were imported into Microsoft Excel 2023 sheets. Statistical analysis was performed using Stata^®^ (Stata 19.0 BE-Basic Edition; StataCorp LLC, Lakeway Drive, College Station, TX, USA), with the statistical significance set at *P* < 0.05. The Chi-square test was used to assess associations between categorical variables, and the independent-samples *t*-test was used to compare the means. For continuous variables, the mean ± SD (and 95% CI when appropriate) was used. Counts (%) were utilized for categorical variables. The Shapiro–Wilk test and Q–Q plots were employed to assess normality, while Levene’s test was utilized to evaluate homogeneity of variance. We utilized Welch’s *t*-test for unequal variances and reported Cohen’s d along with 95% CIs for sex differences. Multivariable linear models were used to analyze canal length and foramen diameters (predictors: sex, age), while multinomial/ordinal logistic models were applied to categorical outcomes (shape, location, angulation). The Benjamini–Hochberg false discovery rate (*q* = 0.05) was used to manage multiple comparisons within families. The two-tailed α was 0.05.

## Results

### Demographic characteristics of the patients

The demographics of the participants involved in this research are provided in (Table 1). Of the 100 patient subjects studied, 53 (53%) were men and 47 (47%) women. This indicates an almost evenly split sex ratio. The age range for the sample was 18–70; approximately fifty percent (49%) of the ages fell into the 21–40-year-old category. This suggests that the participant sample was a diverse group of younger-middle-aged adults with roughly even gender representation. These observations suggest that the study sample consisted of a relatively heterogeneous population, biased toward young to middle-aged adults with near gender parity.

**Table 1:** Demographic characteristics of the respondents.

**Characteristics**	**Frequency (*n*)^a^**	**Percentage (%)**
Male	53	53.0
Female	47	47.0
Total	100	100
18–20 years	10	10.0
21–40 years	49	49.0
41–60 years	29	29.0
61–70 years	12	12.0
Total	100	100

**Notes.**

a *n*, number of patients

### Observer’s reliability and agreement

Inter-observer reliability was assessed using a two-way random intraclass correlation coefficient (ICC = 0.78) for continuous variables, indicating good agreement, while categorical variables showed moderate agreement (weighted kappa = 0.59). This reflects visual categorization rather than metric measurements; therefore, it is unlikely to affect the quantitative results regarding the canal and foramen.

### Sex-based differences in shapes, canal length and foramen diameter

Our results did not reveal any significant association between the shapes of the incisive canal and incisive foramen and sex (Table 2). However, significant sex-based differences were observed in both canal length and buccopalatal foramen diameter (Table 3). The observed mean differences exceeded the minimum detectable thresholds established in the power analysis, supporting the robustness of the findings. As seen in Table 3, males exhibited a significantly longer IC (11.06 ± 2.47 mm) compared to females (9.28 ± 2.68 mm; mean difference = 1.78 mm, *p* = 0.001, Cohen’s *d* = 0.70). Similarly, the buccopalatal diameter of the incisive foramen was significantly larger in males (3.77 ± 1.19 mm) than in females (3.06 ± 0.89 mm; mean difference = 0.71 mm, *p* = 0.001, Cohen’s *d* = 0.67). No significant sex difference was observed for the mesiodistal foramen diameter (*p* = 0.81).

**Table 2:** Associations between the shapes of the incisive canal and incisive foramen and sex.

**Incisive canal shape**	**Male**	**Female**	**Total**	*χ* ^2^	***p*-value**
Hourglass	14	7	21	4.501	0.480
Inverted funnel	6	4	10		
Spindle	9	6	15		
Funnel	13	19	32		
Y	2	1	3		
Cylindrical	9	10	19		
Total	53	47	100		
**Foramen shape**					
Pike	4	3	7	0.754	0.944
Heart	11	10	21		
Clover	4	2	6		
Oval	29	26	55		
Triangle	5	6	11		
Total	53	47	100		

**Table 3:** Sex-based differences in incisive canal length and incisive foramen diameters.

**Parameter**	**Sex**	***N***	**Mean ± SD^a^**	**Mean difference** **(95% CI)**	***t*-test**	***p*-value**	**Cohen’s *d***
Incisive canal length (mm)	Male	53	11.060 ± 2.465	+1.784 (0.77, 2.80)	3.45	0.001^a^	0.70
	Female	47	9.276 ± 2.678				
Foramen diameter (mesiodistal, mm)	Male	53	3.415 ± 0.824	−0.06 (−0.53, 0.42)	0.24	0.81	0.05
	Female	47	3.471 ± 1.462				
Foramen diameter (buccopalatal, mm)	Male	53	3.768 ± 1.187	+0.71 (0.30, 1.12)	3.40	0.001^a^	0.67
	Female	47	3.060 ± 0.889				

**Notes.**

a SD, standard deviation; CI, confidence interval

### Effect of age on foramen diameter

Linear regression analysis was performed to assess the effect of age on foramen diameters (Table 4). Age showed a non-significant association with the mesiodistal diameter of the incisive foramen (*B* = 0.012, SE = 0.007, *p* = 0.088) and with the buccopalatal diameter (*B* = 0.006, SE = 0.007, *p* = 0.401), indicating that foramen size does not vary significantly with age in this sample.

**Table 4:** Linear regression analysis of age versus incisive foramen diameters.

**Model**		**Unstandardized coefficient**		**Standardized coefficient**	**t**	***p*-value**
		***B***	**SE^a^**	**Beta**		
Mesiodistal diameter of the foramen	(Constant)	2.981	0.291		10.234	0.000
	Age	0.012	0.007	0.172	1.724	0.088
Buccopalatal diameter of the foramen	(Constant)	3.217	0.282		11.425	0.000
	Age	0.006	0.007	0.085	0.844	0.401

**Notes.**

a SE, standard error of the mean

### Associations between the position of the IC, presence of additional canals, canal angulation, and age

As shown in Table 5, the position of the IC was not significantly correlated with age (*χ*2 = 7.681, *p* = 0.567). Furthermore, a single canal with a single foramen or a bifurcation, and multiple canals and foramens were the most common variations in all age groups. However, these variations were not significantly related to age (*χ*^2^ = 17.680, *p* = 0.126). Regarding canal angulation (convergence), no statistically significant association with age was observed. Most age groups exhibited mild or moderate convergence, with a few showing almost straight or severe convergence (Table 5).

**Table 5:** Associations between the position of the incisive canal, the presence of additional canals and foramens, the angulation of the canal, and age.

**Description of canal position**	**18–20 y**	**21–40 y**	**41–60 y**	**61–70 y**	**Total**	*χ* ^2^	***p*-value**
<7 mm	2	8	1	2	13	7.681	0.567
7–9 mm	2	12	9	6	29		
10–12 mm	4	15	9	3	31		
>12 mm	2	13	9	1	25		
Total	10	48	28	12	98		
Number of incisive canals and foramens							
Single canal with a single foramen	6	32	12	8	58	17.680	0.126
Single canal with multiple foramens	3	12	7	1	23		
Two parallel canals and foramens	0	0	2	0	2		
Multiple canals and foramens	1	0	1	0	2		
Two canals, one foramen	0	5	7	3	15		
Total	10	49	29	12	100		
Canal angulation							
Almost straight	3	8	7	1	19	5.144	0.822
Mild	4	22	10	6	42		
Moderate	2	18	10	4	34		
Almost severe	1	1	2	1	5		
Total	10	49	29	12	100		

**Notes.**

The canal location assessments were based on a sample size of *n* = 98 due to insufficient coronal coverage in two scans.

### Associations between the position of the IC, the presence of additional canals, canal angulation, and sex

Table 6 shows the distribution of canal extension beyond the nasal floor among male and female patients. The total number of patients examined in this experiment was 98, as two CBCT scans lacked adequate coronal coverage. The 10–12 mm category was the most frequent overall (*n* = 31). In males, extensions greater than 12 mm predominated (*n* = 22), whereas in females most canals extended 7–9 mm below the nasal floor (*n* = 18). (*χ*^2^ = 17.54, *p* = 0.001). The distribution of various canal and foramen types between males and females were not significantly different (*χ*^2^ = 2.37, *p* = 0.666). In general, anatomic variations of the canal and foramen were observed, but no significant effects of sex were detected. The most frequently observed patterns in males and females were mild and moderate convergence, whereas almost straight and severe forms occurred less frequently (Table 6).

**Table 6:** Associations between the position of the incisive canal, the presence of additional canals and foramens, the angulation of the canal, and sex.

**Canal extension beyond the nasal floor**	**Male**	**Female**	**Total**	*χ* ^2^	***P*-value**
<7 mm	4	9	13	17.549	0.001^a^
7–9 mm	11	18	29		
10–12 mm	16	15	31		
>12 mm	22	3	25		
Total	53	45	98		
Number of incisive canals and foramens					
Single canal with a single foramen	32	26	58	2.379	0.666
Single canal with multiple foramens	12	11	23		
Two parallel canals and foramens	0	2	2		
Multiple canals and foramens	1	1	2		
Two canals, one foramen	8	7	15		
Total	53	47	100		
Canal angulation					
Almost straight	9	10	19	2.754	0.431
Mild	26	16	42		
Moderate	15	19	34		
Almost severe	3	2	5		
Total	53	47	100		

**Notes.**

The canal location assessments were based on a sample size of *n* = 98 due to insufficient coronal coverage in two scans.

## Discussion

Although traditionally regarded as a remnant of the nasopalatine duct, conflicting reports suggest that the IC may instead be a bony canal arising during palatogenesis (Shylaja, Balaji & Krishna, 2011; Lake et al., 2018). Observed variations in the morphology and position of the IC and foramen further support the idea that their development is not uniform, underscoring the need for additional research to clarify their embryological origin and clinical implications. CBCT studies have enhanced our understanding of anatomical and morphometric variations in the IC, influenced by factors such as age, sex, ethnicity, tooth loss, and trauma (Thakur et al., 2013; Özeren Keşkek, Aytuğar & Çene, 2022; Alhumaidi et al., 2024). To the best of our knowledge, this is the first study to investigate these aspects in a population from the Asir region of Saudi Arabia, particularly Abha City residents visiting King Khalid University. While previous CBCT studies have described IC morphology, variability related to geographic and demographic factors necessitates population-specific evaluation. The current study addresses this gap by providing detailed morphometric data from the Asir region of Saudi Arabia, thereby enhancing the clinical applicability of anatomical reference values for local implant practice.

The shape of the IC has been classified differently depending on imaging planes and studies. On axial slices, six types were described based on shape and separation—round, oval, and heart-shaped, with or without separation (Acar & Kamburoğlu, 2015). Another study categorized the shape of the IC into six groups: tree branch, cylinder, banana, funnel, cone, and hourglass (Etoz & Sisman, 2014). In sagittal views, classifications range from four to nine types, including funnel, cylindrical, hourglass, banana-like, cone, spindle, tree branch-like, and others (Sekerci, Buyuk & Cantekin, 2014; Jain et al., 2017). Some studies reported only four forms: cylindrical, funnel, spindle, and hourglass (Safi et al., 2017; Nasseh, Aoun & Sokhn, 2017). These variations likely reflect differences in imaging technique, anatomical diversity, and developmental factors, underscoring the complexity of the IC’s morphology. In the current study, IC shape was divided into six types: hourglass, inverted funnel, spindle, funnel, Y-shape, and cylindrical. As in previous studies (Fukuda et al., 2015; Jayasinghe et al., 2019), the funnel shape was the most common, followed by the hourglass and cylindrical shapes, with the Y-shape being the least common. The primary shape of the foramen in the current study was oval, followed by a heart-shaped form. As reported previously (Jayasinghe et al., 2019; Piskórz et al., 2024), the shape of the IC was not correlated with sex.

The mean length of the IC in this study was significantly higher in males (11.06 mm) than in females (9.28 mm), a finding similar to those reported in previous studies (Tözüm et al., 2012; Thambi & Aswath, 2024; Piskórz et al., 2024), which could be due to differences in craniofacial size and bone structure. Variations in IC morphology attributed to differences in ethnicity, age-related changes, and environmental factors have been reported previously (Milanovic et al., 2021; Firincioglulari & Orhan, 2024). Consistent with previous studies (Soumya et al., 2019; Devadiga et al., 2025), the mean mesiodistal and buccopalatal diameters of the foramen in this study were 3.44 mm and 3.44 mm, respectively. The mesiodistal diameter of the foramen was not correlated with sex but showed a slight tendency to increase with age (Table 4). In contrast, the buccopalatal diameter differed significantly by sex, but not by age.

Regarding the relationship between incisive foramen diameter and sex, some studies have reported that males tend to have larger incisive foramen diameters than females (Devadiga et al., 2025), possibly due to overall differences in craniofacial size and bone structure. Others found no significant age-related differences in terms of the dimensions of the IC (Yadav et al., 2024), suggesting that variations may be influenced more by individual anatomical diversity. These conflicting observations emphasize the importance of considering both sex and other factors, such as age and ethnicity, when evaluating the IC and incisive foramen in clinical practice. Regarding the position of the IC, extensions exceeding 12 mm were more frequent in males, and shorter canals (<7 mm) were more common in females; no significant associations with age were observed, indicating that sex has a greater influence on canal position than age. Similar to previous reports (Thakur et al., 2013; Al-Amery et al., 2015; Safi et al., 2017; Jayasinghe et al., 2019), most of the patients in the present study had a single canal with one (58%) or two (23%) foramens; however, statistically significant differences between genders with respect to the number of additional canals and foramens were not observed.

Mild IC angulation (in males) was most prevalent in this study, followed by moderate angulation (in females). Moreover, these two angulations were most common in the 21–60-year age groups. Higher IC angulations have been reported in males compared to females in previous studies (Safi et al., 2017; Devadiga et al., 2025). Precise identification of these variations is vital to avoid damaging the neurovascular contents of the IC, and CBCT can facilitate their detection and prevention.

The recognized sexual dimorphisms in canal length and buccopalatal foramen diameter have substantial implications for planning implant trajectories in the anterior maxilla, where proximity to the nasopalatine neurovascular bundle increases the risks of hemorrhage, neurosensory disturbances (paresthesia), and implant failure (Mraiwa et al., 2004; Bornstein et al., 2011; Tözüm et al., 2012). In addition to implantology, the description of canal positioning in relation to the nasal floor determines the safety of orthodontic mini-implants and affects periodontal surgical techniques, such as palatal flap design. The presence of modest to moderate angulation helps predict canal curvature when creating guides and selecting anesthetic techniques.

In the different regions of Saudi Arabia, several CBCT-based analyses of the IC and incisive foramen have been reported. Our findings revealed that funnel shape followed by hourglass and cylindrical, are the most frequent shapes, and the oval shape, followed by heart shape, is the most frequent foramen shape. On the contrary, Alasmari (2023) stated that the cone-shaped canal is the most frequent, followed by cylindrical and hourglass-shaped canals, indicating regional variations. Regarding IC size, Alasmari (2023) reported an overall length of 13.86 ± 2.68 mm while the IC length reported in our study was significantly higher in males compared to females (11.06 mm and 9.28 mm, respectively). In accordance with our findings, Alasmari (2023) and Linjawi et al. (2021) reported greater canal dimensions in males compared to females. Furthermore, Linjawi et al. pointed out that there is no significant association with age, which aligns with our finding of largely similar measurements across age groups. Although Alshamrani, Tokhtah & Al-Omar (2021) evaluated the mandibular incisive canal (MIC) instead of IC, their study from Riyadh reported gender-based dimensional variations, supporting the idea of sexual dimorphism in craniofacial canal morphology in Saudi cohorts.

This study has some limitations. First, this was a single-center cross-sectional analysis of clinically indicated CBCT scans from residents of Abha City (Asir area); therefore, the findings may not be fully representative of the broader Saudi population. Second, although voxel and slice parameters were standardized for acquisition and reporting, morphometry accuracy may vary due to resolution and the presence of artifacts. Third, scans in which the IC was not clearly visualized were excluded, potentially leading to a slight overestimation of mean canal dimensions. However, the exclusion was based on inadequate visualization for reliable measurement, and including poorly defined canals could have increased measurement error. The 0.20-mm voxel size represents a practical resolution limit for detecting extremely small structures. Fourth, the dental status was not evaluated in this study. As alveolar ridge resorption following tooth loss may alter the apparent length and position of the IC, particularly in edentulous individuals, this factor may have influenced age-related findings. Future studies should account for anterior dental status to better isolate anatomical variations from secondary changes related to tooth loss. Consequently, the interpretation of the results must be taken carefully, as future studies are advised to use larger, prospectively collected, and age-balanced samples from multiple centers.

## Conclusions

This study highlights variations in the morphometric characteristics, angles, shapes, dimensions, and positions of the IC and foramen across different age groups and sexes among Abha City residents in Saudi Arabia, using CBCT. Males exhibited greater canal length and buccopalatal foramen diameter compared to females. In contrast, most morphometric characteristics were largely similar across age groups, indicating limited age-related variation within this population. The greater canal dimensions observed in males may influence available bone volume and implant trajectory in the anterior maxilla, potentially increasing proximity to the nasopalatine neurovascular bundle. These findings underscore the importance of individualized CBCT-based preoperative assessment to optimize implant planning and minimize surgical complications. Furthermore, the region-specific findings contribute to the existing CBCT literature by providing population-based reference data on the nasopalatine region among Abha City residents in Saudi Arabia.

##  Supplemental Information

10.7717/peerj.21701/supp-1 Supplemental Information 1 STROBE Checklist

10.7717/peerj.21701/supp-2 Supplemental Information 2 Raw data

10.7717/peerj.21701/supp-3 Supplemental Information 3 Study flow diagram

## Abbreviations

CBCT: Cone-beam computed tomography
IC: Incisive canal

## Additional Information and Declarations

### Data Availability

The following information was supplied regarding data availability: The raw data is available in the Supplemental Files.
