# Supplementary material for: Morphometric analysis of the incisive canal and foramen using CBCT: a cross-sectional study of the Saudi subpopulation
Source: PeerJ. 2026 Sep 28;14:e21701. doi: 10.7717/peerj.21701 (PMC13629566; doi:10.7717/peerj.21701)
Supplement: Supplementary file 1 [file peerj-14-21701-s003.docx]

**Participants flow diagram**

Reason for exclusion:

1- Poor image quality
2- Limited field of view
3- Image artifact
4- Images for patients <18 years old

Reason for inclusion:

1- Diagnostically acceptable CBCT
2- Field of view covering the anterior maxilla
3- CBCT of patients >18 years old

Excluded=100

Included for the final analysis = 100

CBCT scans screened initially = 200
