# Supplementary material for: Morphometric analysis of the incisive canal and foramen using CBCT: a cross-sectional study of the Saudi subpopulation
Source: PeerJ. 2026 Sep 28;14:e21701. doi: 10.7717/peerj.21701 (PMC13629566; doi:10.7717/peerj.21701)
Supplement: Supplementary file 2 [file peerj-14-21701-s001.doc]

STROBE Statement—Checklist of items that should be included in reports of ***cross-sectional studies***

|  | Item No | Recommendation |
| --- | --- | --- |
| **Title and abstract** | 1 | (*a*) Indicate the study’s design with a commonly used term in the title or the abstract  Line 2 |
|  |  | (*b*) Provide in the abstract an informative and balanced summary of what was done and what was found  Lines 17-45 |
| Introduction | | |
| Background/rationale | 2 | Explain the scientific background and rationale for the investigation being reported  Lines 79-97 |
| Objectives | 3 | State specific objectives, including any prespecified hypotheses  Lines 93-97 |
| Methods | | |
| Study design | 4 | Present key elements of study design early in the paper  Lines 100-112 |
| Setting | 5 | Describe the setting, locations, and relevant dates, including periods of recruitment, exposure, follow-up, and data collection  Lines 101-102, lines 105-16 |
| Participants | 6 | (*a*) Give the eligibility criteria, and the sources and methods of selection of participants  Lines 108-112 |
| Variables | 7 | Clearly define all outcomes, exposures, predictors, potential confounders, and effect modifiers. Give diagnostic criteria, if applicable  Lines 137, 147, 162 |
| Data sources/ measurement | 8* | For each variable of interest, give sources of data and details of methods of assessment (measurement). Describe comparability of assessment methods if there is more than one group  Lines 132-176 |
| Bias | 9 | Describe any efforts to address potential sources of bias  Lines 106-108, lines 159-161 |
| Study size | 10 | Explain how the study size was arrived at  Lines 123-132 |
| Quantitative variables | 11 | Explain how quantitative variables were handled in the analyses. If applicable, describe which groupings were chosen and why  Lines 155-159, lines 166-170 |
| Statistical methods | 12 | (*a*) Describe all statistical methods, including those used to control for confounding  Lines 188-192 |
|  |  | (*b*) Describe any methods used to examine subgroups and interactions  Age and sex were described as predictors line 189. No subgroup analysis was performed |
|  |  | (*c*) Explain how missing data were addressed  Line 179 |
|  |  | (*d*) If applicable, describe analytical methods taking account of sampling strategy  Not applicable |
|  |  | (*e*) Describe any sensitivity analyses  Not performed |
| Results | | |
| Participants | 13* | (a) Report numbers of individuals at each stage of study—eg numbers potentially eligible, examined for eligibility, confirmed eligible, included in the study, completing follow-up, and analysed  Line 196 |
|  |  | (b) Give reasons for non-participation at each stage  Lines 111-113 |
|  |  | (c) Consider use of a flow diagram  The diagram is provided as supplementary |
| Descriptive data | 14* | (a) Give characteristics of study participants (eg demographic, clinical, social) and information on exposures and potential confounders  Line 197 |
|  |  | (b) Indicate number of participants with missing data for each variable of interest  Line 232 |
| Outcome data | 15* | Report numbers of outcome events or summary measures  Lines 204-240 |
| Main results | 16 | (*a*) Give unadjusted estimates and, if applicable, confounder-adjusted estimates and their precision (eg, 95% confidence interval). Make clear which confounders were adjusted for and why they were included  Table 3-6 |
|  |  | (*b*) Report category boundaries when continuous variables were categorized  Lines 156-159, lines 167-170 |
|  |  | (*c*) If relevant, consider translating estimates of relative risk into absolute risk for a meaningful time period  Not applicable |
| Other analyses | 17 | Report other analyses done—eg analyses of subgroups and interactions, and sensitivity analyses  Lines 195-203 |
| Discussion | | |
| Key results | 18 | Summarise key results with reference to study objectives  Lines 242-254 |
| Limitations | 19 | Discuss limitations of the study, taking into account sources of potential bias or imprecision. Discuss both direction and magnitude of any potential bias  317-322 |
| Interpretation | 20 | Give a cautious overall interpretation of results considering objectives, limitations, multiplicity of analyses, results from similar studies, and other relevant evidence  Lines 321-323 |
| Generalisability | 21 | Discuss the generalisability (external validity) of the study results  Line 317-318 |
| Other information | | |
| Funding | 22 | Give the source of funding and the role of the funders for the present study and, if applicable, for the original study on which the present article is based  Lines 335-338 |

*Give information separately for exposed and unexposed groups.

**Note:** An Explanation and Elaboration article discusses each checklist item and gives methodological background and published examples of transparent reporting. The STROBE checklist is best used in conjunction with this article (freely available on the Web sites of PLoS Medicine at http://www.plosmedicine.org/, Annals of Internal Medicine at http://www.annals.org/, and Epidemiology at http://www.epidem.com/). Information on the STROBE Initiative is available at www.strobe-statement.org.
